# Supplementary material for: Nanopore adaptive sampling: a tool for enrichment of low abundance species in metagenomic samples
Source: Genome Biol. 2022 Jan 24;23:11. doi: 10.1186/s13059-021-02582-x (PMC8785595; doi:10.1186/s13059-021-02582-x)
Supplement: Supplementary file 2 — Additional file 2. DNA quantification. Figures S1-S17 showing TapeStation and Femto Pulse traces for bacterial DNA extractions. [file 13059_2021_2582_MOESM2_ESM.pdf]

Additional File 2: DNA quantification

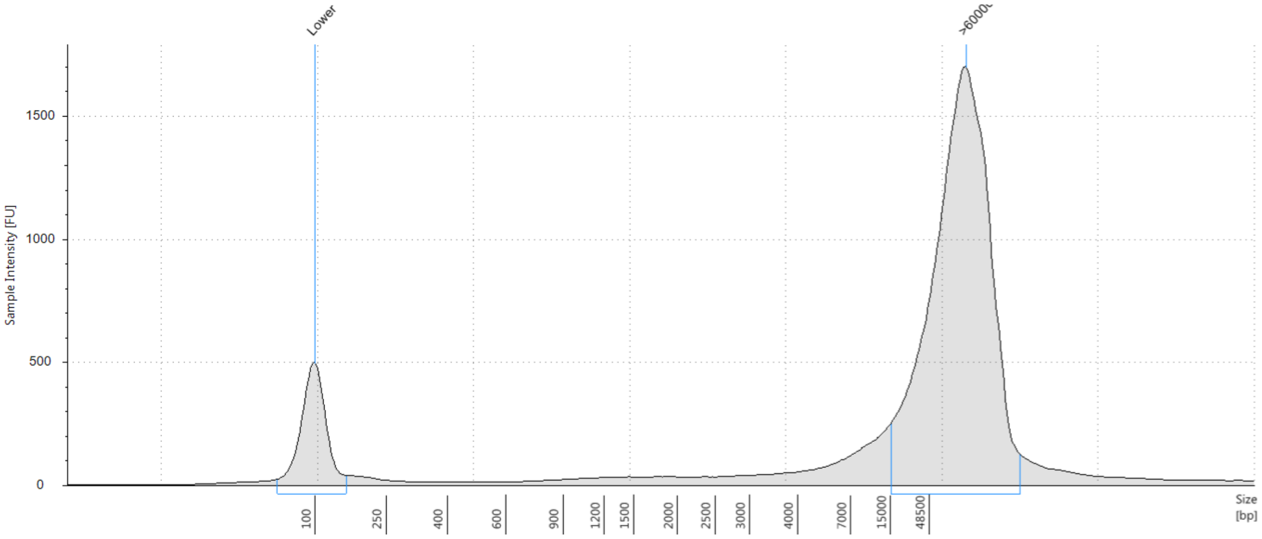

Figure S1: *Achromobacter xylosoxidans* extraction Genomic TapeStation trace. DNA used for 1.7kbp, 4.7kbp, 12.8kb runs.

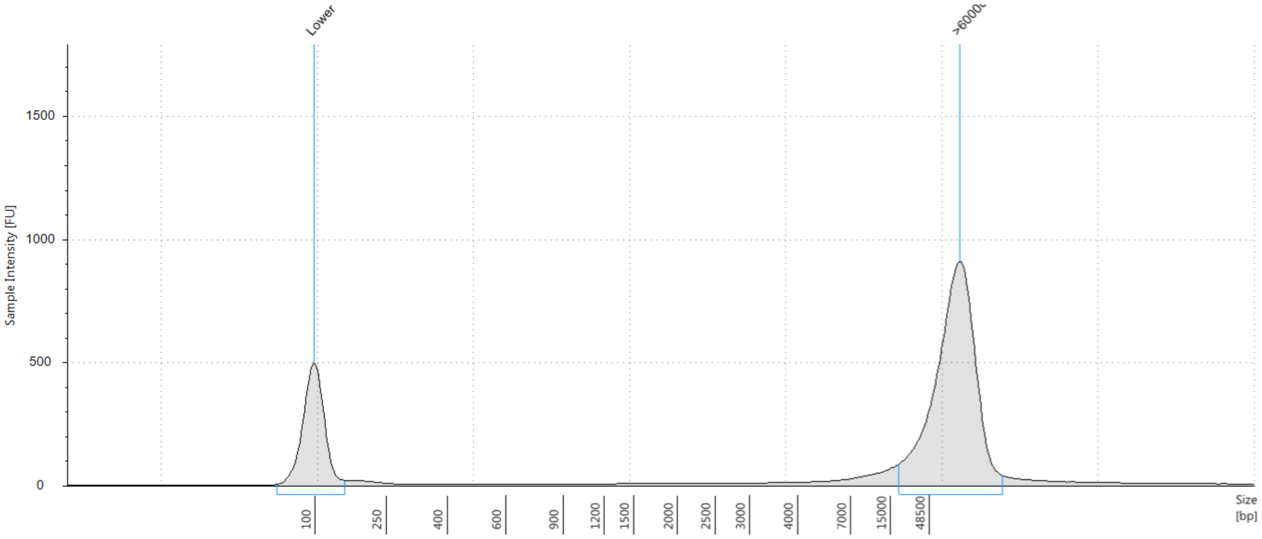

Figure S2: *Morganella morganii* extraction Genomic TapeStation trace. DNA used for 1.7kbp, 4.7kbp, 12.8kb runs.

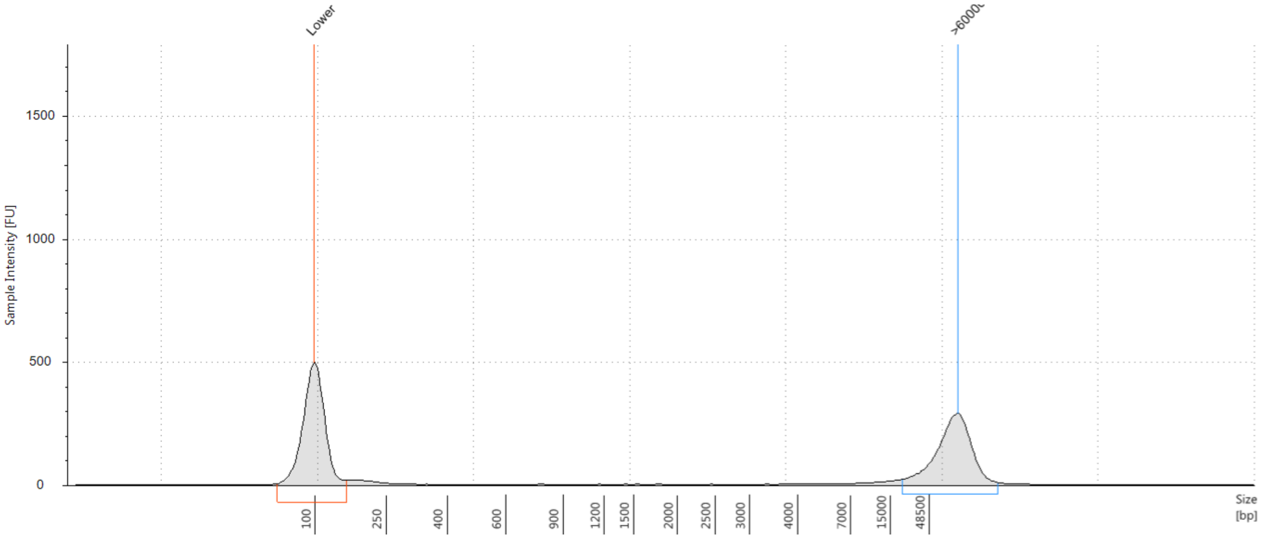

Figure S3: *Leminorella richardii* extraction Genomic TapeStation trace. DNA used for 1.7kbp, 4.7kbp, 12.8kb runs.

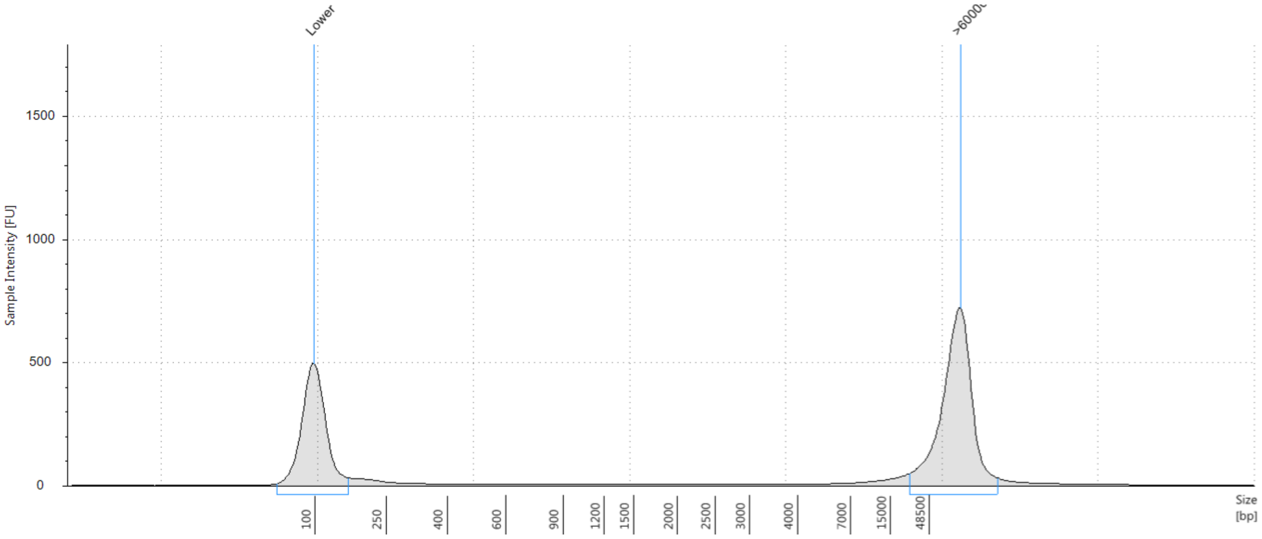

Figure S4: *Moellerella wisconsensis* extraction Genomic TapeStation trace. DNA used for 1.7kbp, 4.7kbp, 12.8kb runs.

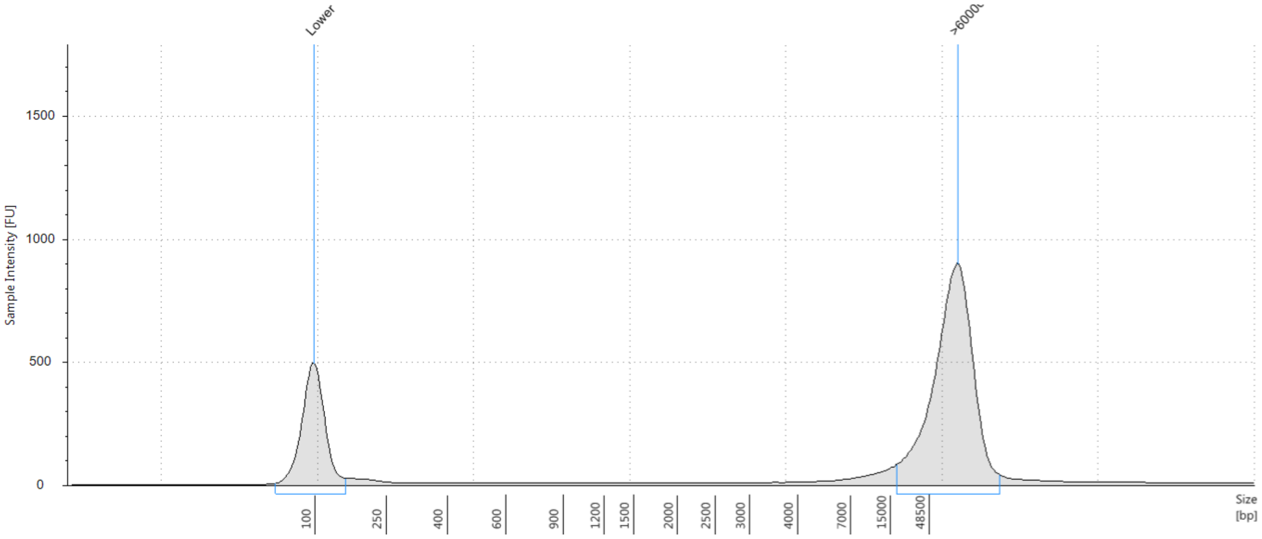

Figure S5: *Pseudomonas aeruginosa* extraction Genomic TapeStation trace. DNA used for 1.7kbp, 4.7kbp, 12.8kb runs.

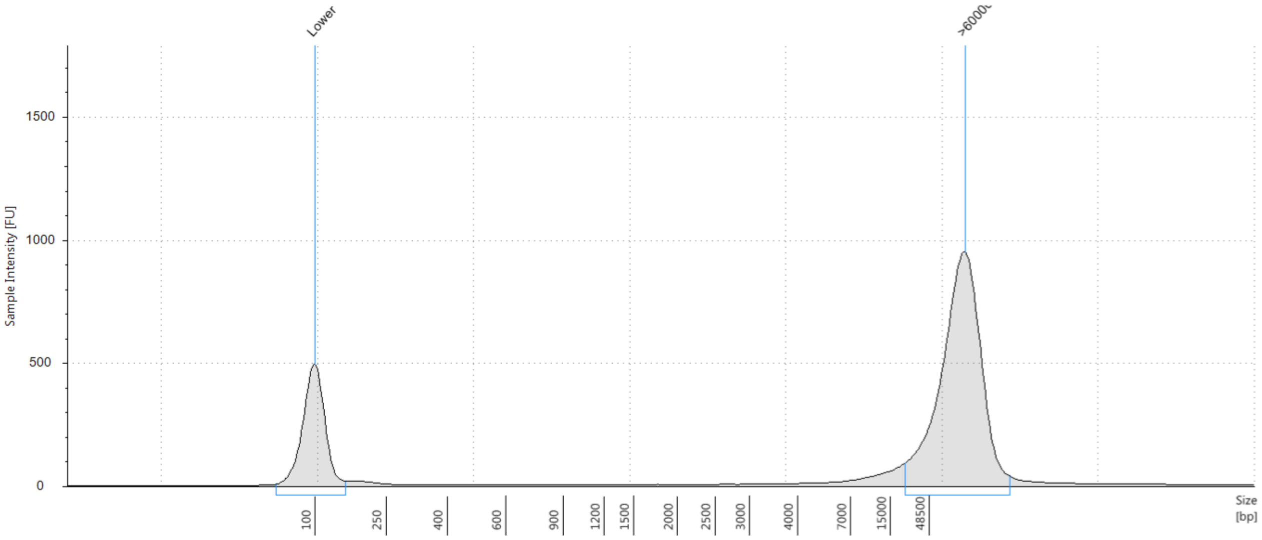

Figure S6: *Proteus vulgaris* extraction Genomic TapeStation trace. DNA used for 1.7kbp, 4.7kbp, 12.8kb runs.

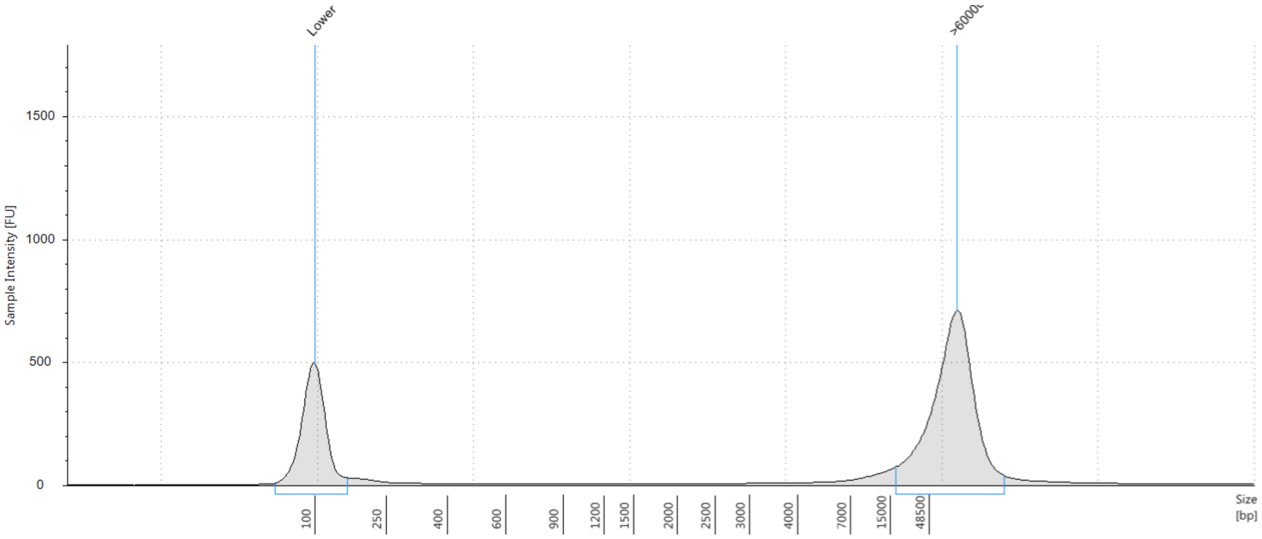

Figure S7: *Streptococcus dysgalactiae* extraction Genomic TapeStation trace. DNA used for 1.7kbp, 4.7kbp, 12.8kb runs.

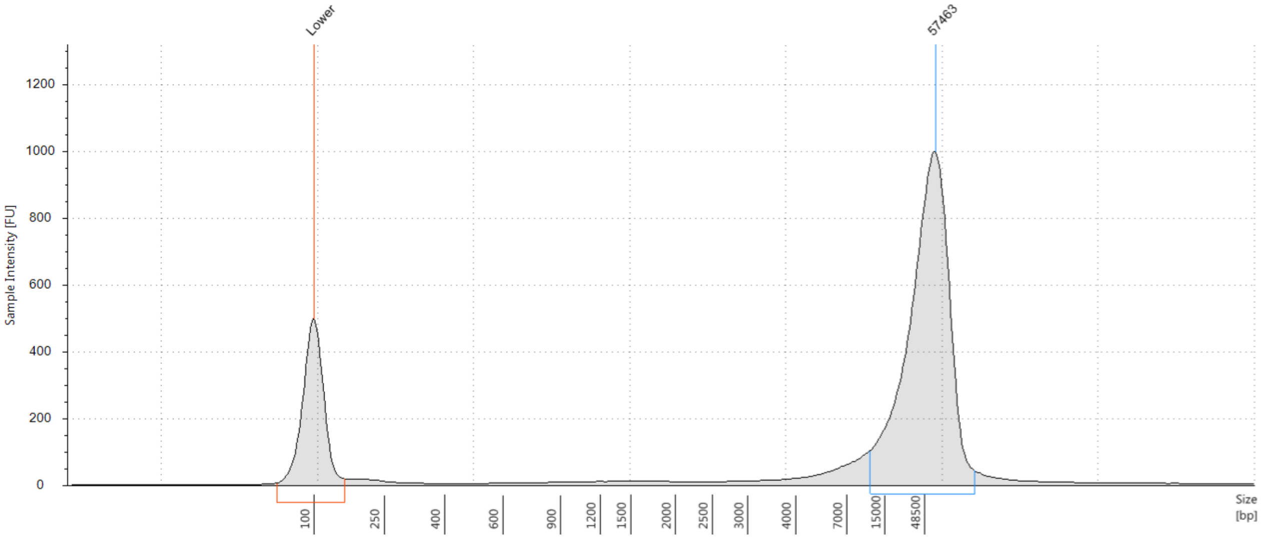

Figure S8: Unfragmented mock Genomic TapeStation trace. DNA used for 1.7kbp, 4.7kbp, 12.8kb runs.

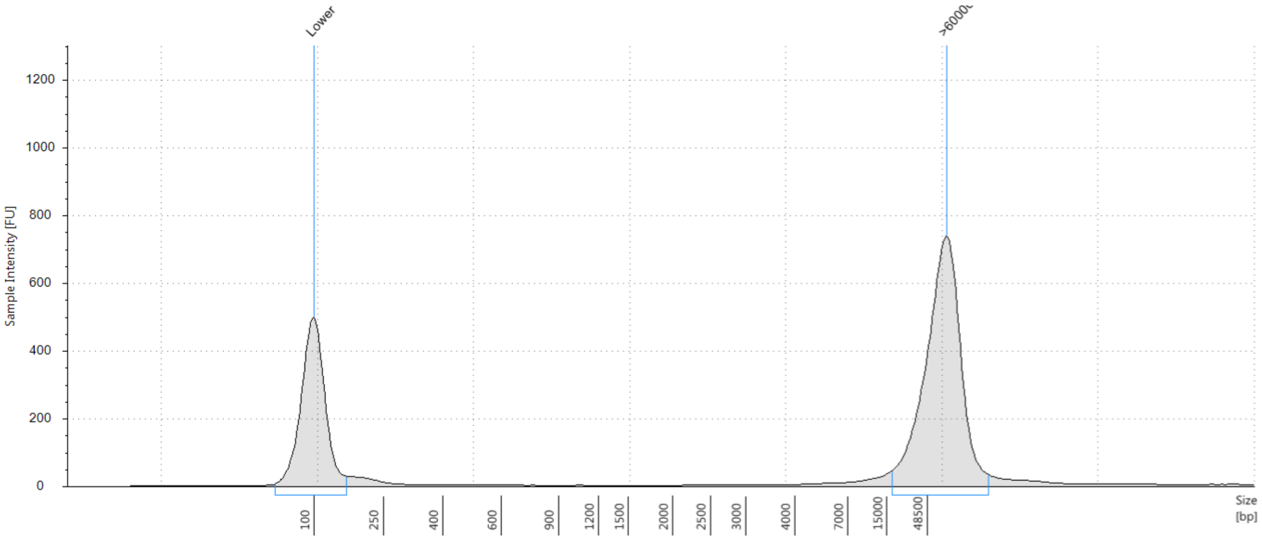

Figure S9: 12.8kbp mean fragmented library Genomic TapeStation trace. DNA used for 1.7kbp, 4.7kbp, 12.8kb runs.

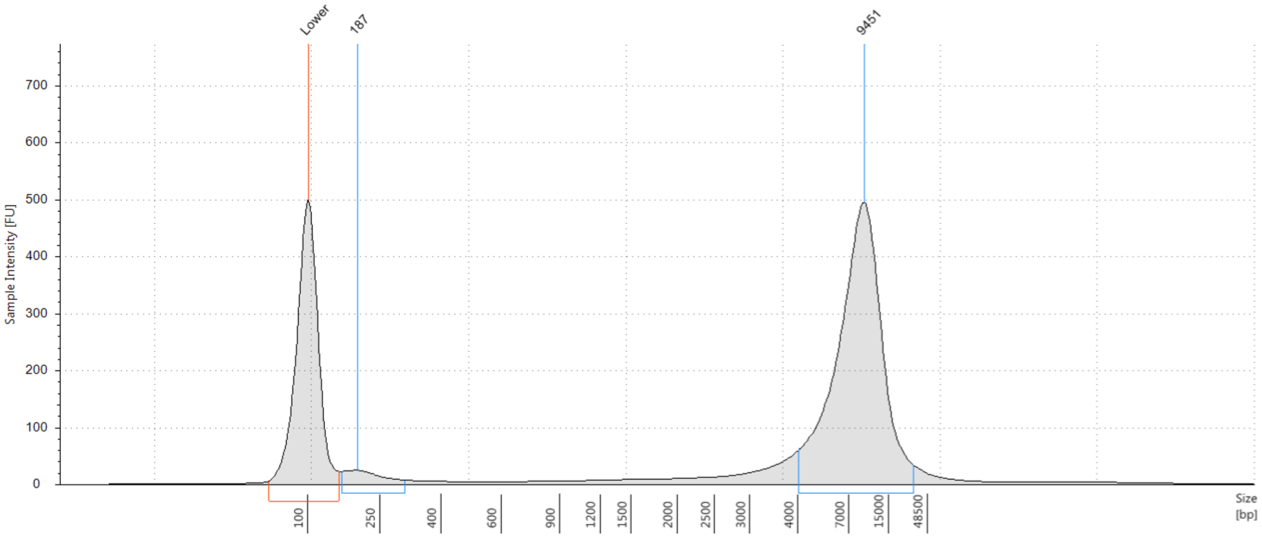

Figure S10: 4.7kbp mean fragmented library Genomic TapeStation trace. DNA used for 1.7kbp, 4.7kbp, 12.8kb runs.

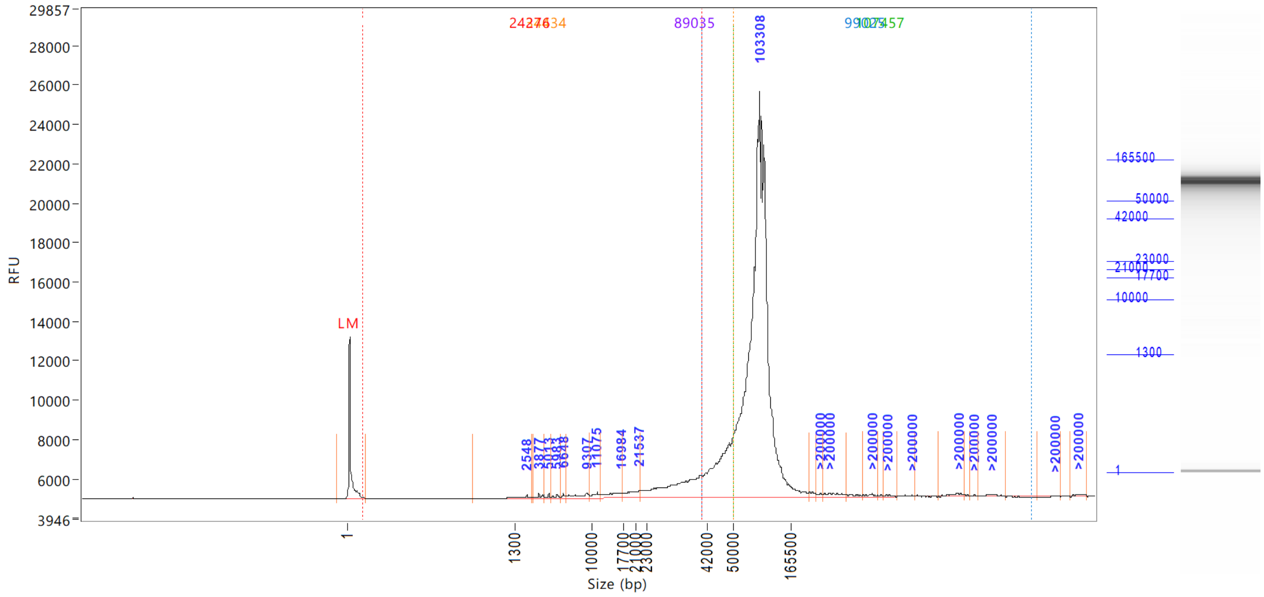

Figure S11: *Achromobacter xylosoxidans* extraction Femto Pulse trace. DNA used for 10.6 kbp runs.

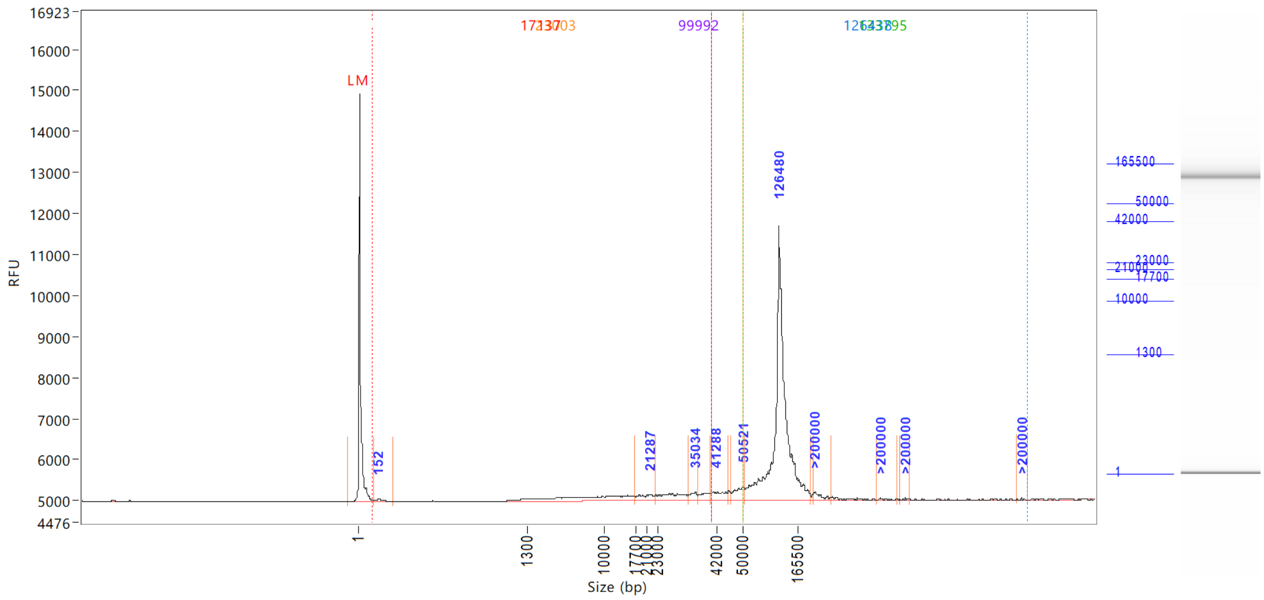

Figure S12: *Morganella morganii* extraction Femto Pulse trace. DNA used for 10.6 kbp runs.

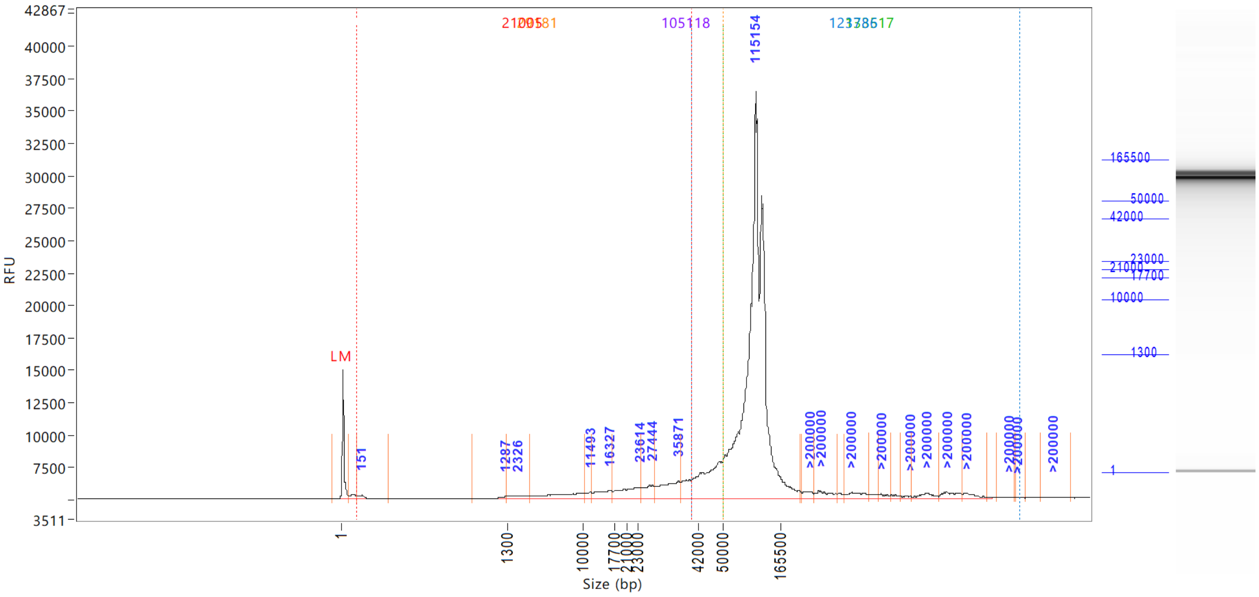

Figure S13: *Leminorella richardii* extraction Femto Pulse trace. DNA used for 10.6 kbp runs.

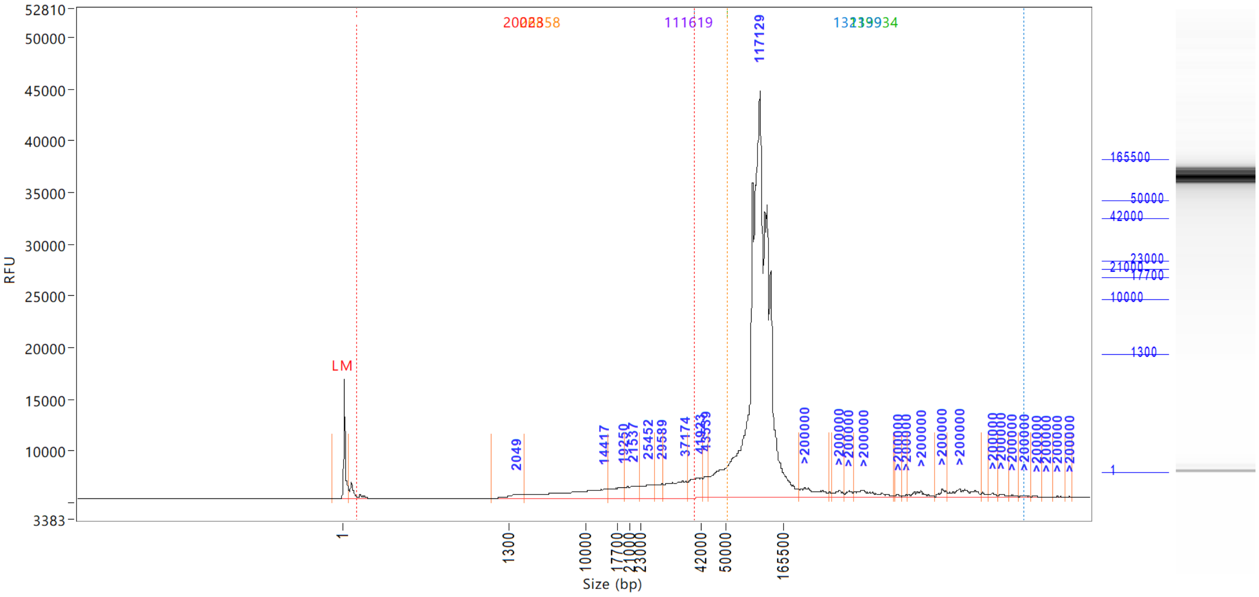

Figure S14: *Moellerella wisconsensis* extraction Femto Pulse trace. DNA used for 10.6 kbp runs.

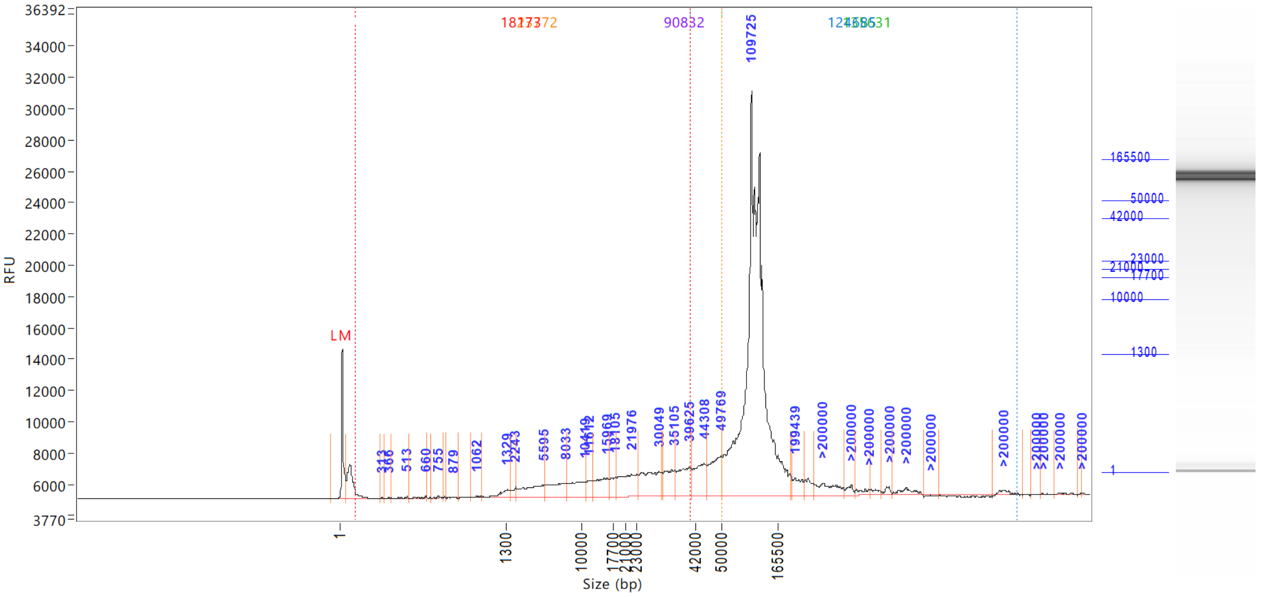

Figure S15: *Pseudomonas aeruginosa* extraction Femto Pulse trace. DNA used for 10.6 kbp runs.

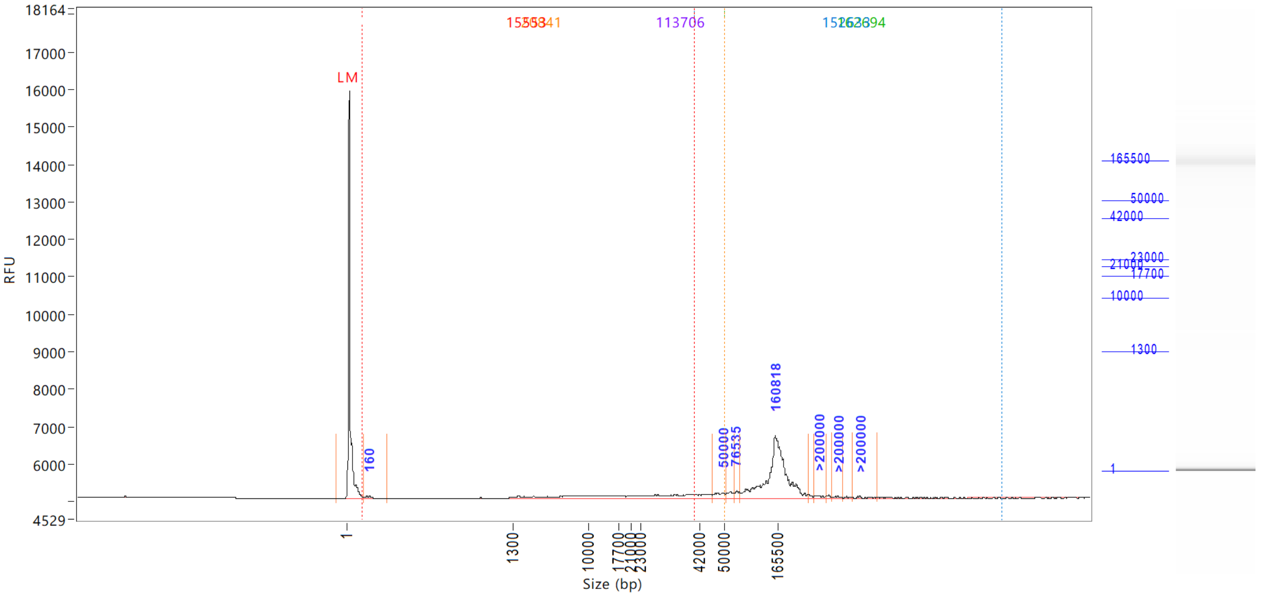

Figure S16: *Proteus vulgaris* extraction Femto Pulse trace. DNA used for 10.6 kbp runs.

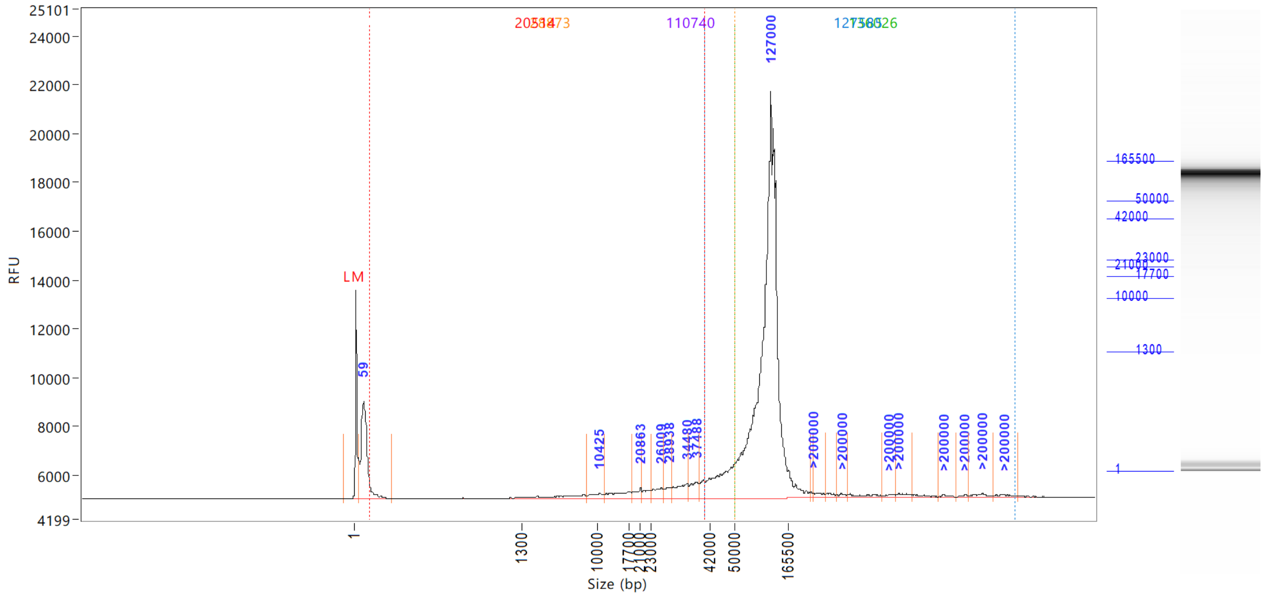

Figure S17: *Streptococcus dysgalactiae* extraction Femto Pulse trace. DNA used for 10.6 kbp runs.
