# Supplementary material for: Nanopore adaptive sampling: a tool for enrichment of low abundance species in metagenomic samples
Source: Genome Biol. 2022 Jan 24;23:11. doi: 10.1186/s13059-021-02582-x (PMC8785595; doi:10.1186/s13059-021-02582-x)
Supplement: Supplementary file 3 — Additional file 3. Review history [file 13059_2021_2582_MOESM3_ESM.docx]

Review History

**First round of review**

**Reviewer 1**

**Were you able to assess all statistics in the manuscript, including the appropriateness of statistical tests used?**

Yes, and I have assessed the statistics in my report.

**Comments to author:**

The article by Martin et al illustrates the use of ONT adaptive sampling applied to metagenomics. The authors have developed a mathematical model that allows the user to estimate the benefit of using adaptive sampling, based on an estimated species abundance. Theoretically, this offers a very elegant solution for trying to access low abundant species, or to improve the assembly of a specific species. As the authors appropriately highlight, the method is best used on higher average molecule lengths.

The authors have provide a clear explanation of how they have developed and benchmarked their model using a mock community. I do not have any fundamental issues with this mock community, but the model is simplistic. One of the uses cases that the authors wish to address is to improve MAG quality. A fundamental cause of chimeric MAGs is when two organisms share a region of DNA. This could be from the fact these organisms are closely related or from horizontally transferred elements (e.g. phage). In the adaptive sampling, I would assume that a degree of relatedness is going to play a key role in the success of the experimental approach? The authors have described false negatives, but this would surely lead to false positives, and a reduction in the enrichment process. To understand this aspect, the authors need to construct additional mock communities where there are multiple related organisms and where the number of organisms included is increased. With known genomes, the reference database for enrichment/depletion can be adapted appropriately, however, for the majority of metagenomic samples, this will not be the case. Thus, it would greatly strengthen the manuscript if the authors were to demonstrate the adaptive sampling on real metagenomics samples, such as the human gut samples. On a related note, it would be interesting for the authors to provide a commentary on the ability to enrich specific strains. MAGs often represent a population encompassing multiple strains. Nevertheless, despite these reservations I consider the methodological approach to be interesting and provide another experimental avenue for untangling microbial communities. Just in the present for, I feel the work is incomplete for me to be persuaded that I should try this on my samples.

Minor

The introduction/background contained a very thorough overview of different enrichment strategies. While informative, I felt that some of the content was becoming off-topic, especially the paragraph describing the exonic probe sets.

It would be beneficial to include a description of how the GridION adaptive sampling is implemented in the introduction. While the authors describe the requirements, there are no details on how the sequences are actually used.

There are a few places in the manuscript where there is the inappropriate contraction of words, e.g. "can't" 397, page 16

**Reviewer 2**

**Were you able to assess all statistics in the manuscript, including the appropriateness of statistical tests used?**

Yes, and I have assessed the statistics in my report.

**Comments to author:**

This manuscript comprises both an evaluation of Nanopore adaptive sequencing in the context of bacterial metagenomics and the introduction and testing of a simple mathematical model to predict the degree of enrichment of any species in a community. Nanopore adaptive sequencing is an appealing technology in the context of metagenomics for the reasons outlined by the authors. In mixed communities being able to select for a species or strain of interest has numerous applications. The authors do not introduce a new bioinformatics strategy for doing this but instead evaluate the ONT algorithm. The major novelty in the study lies in the model and the results of testing seven species mock community algorithm which was tested over a range of fragment lengths.

The model is simple, being quite similar to the derivation of Michaelis-Menten kinetics for enzymes but appears to be very accurate. I believe that people performing such studies will find being able to predict the degree of enrichment of the target species to be very useful. I was less impressed by the mock community experiments themselves, seven species is not a complex community and may not be very representative of real samples not least because there was no strain diversity. Given that a lot of the value of this paper lies in the mock analysis I think a more realistic community would have been better. Furthermore, the authors worked from the assumption that the exact reference sequence of the strain of interest will be available. This is an unrealistic assumption. Many applications of the technique may consist of amplifying a species without knowing the precise strain that may be present. The authors could have explored this by including only genomes from related strains of the same species in the reference database. Or even if a larger taxonomic is targeted, for instance enriching for fungi in general would be very relevant in microbiome studies. It would have also added greatly to the paper if they had actually applied the method to a real sample in a real application context.

Some minor points are that I found the introduction a bit unfocussed the discussion of MAGs did not seem the obvious way to start a motivation of the method and the digression into RNA sequence also seemed a bit superfluous. It was also unclear what method of sequence comparison is used in the program tested here but that be because the details are not revealed by ONT.

On balance whilst I believe the model is practical and of interest, the actual mock community results as performed do not sufficiently explore the real limitations of the method or clearly demonstrate practical uses of the methodology.

**Authors Response**

**Point-by-point responses to the reviewers’ comments:**

Reviewer 1:

The article by Martin et al illustrates the use of ONT adaptive sampling applied to metagenomics. The authors have developed a mathematical model that allows the user to estimate the benefit of using adaptive sampling, based on an estimated species abundance. Theoretically, this offers a very elegant solution for trying to access low abundant species, or to improve the assembly of a specific species. As the authors appropriately highlight, the method is best used on higher average molecule lengths.

*We thank the reviewer for taking the time to read the manuscript and for their helpful comments. We hope we have been able to address all their comments in this new revision.*

The authors have provide a clear explanation of how they have developed and benchmarked their model using a mock community. I do not have any fundamental issues with this mock community, but the model is simplistic. One of the uses cases that the authors wish to address is to improve MAG quality. A fundamental cause of chimeric MAGs is when two organisms share a region of DNA. This could be from the fact these organisms are closely related or from horizontally transferred elements (e.g. phage). In the adaptive sampling, I would assume that a degree of relatedness is going to play a key role in the success of the experimental approach? The authors have described false negatives, but this would surely lead to false positives, and a reduction in the enrichment process. To understand this aspect, the authors need to construct additional mock communities where there are multiple related organisms and where the number of organisms included is increased. With known genomes, the reference database for enrichment/depletion can be adapted appropriately, however, for the majority of metagenomic samples, this will not be the case. Thus, it would greatly strengthen the manuscript if the authors were to demonstrate the adaptive sampling on real metagenomics samples, such as the human gut samples. On a related note, it would be interesting for the authors to provide a commentary on the ability to enrich specific strains. MAGs often represent a population encompassing multiple strains. Nevertheless, despite these reservations I consider the methodological approach to be interesting and provide another experimental avenue for untangling microbial communities. Just in the present for, I feel the work is incomplete for me to be persuaded that I should try this on my samples.

*Our original aim with this paper was to provide a clear understanding of what might be possible when utilising the adaptive sampling approach on metagenomic samples. As a result, our focus was on exploring our model and showing how the variables affect the potential levels of enrichment. However, the reviewer highlights some interesting questions and we have attempted to shed light on these in the revised paper. To that end, we have sequenced two additional samples using adaptive sequencing. In the first, we sequence a more complex gut microbial standard that includes 5 strains of E. coli. In the second, we sequence compost, a complex real-world sample. We show that enriching for a single strain of E. coli ends up enriching the other 4 strains as well. The desirability of this depends on the particular experiment being performed - sometimes this will be a problem, sometimes a benefit. We also show that good enrichment is achievable from the compost using both a previously published reference genome and a genome assembled from the same metagenomic sample, and compare the use of these different references.*

Minor

The introduction/background contained a very thorough overview of different enrichment strategies. While informative, I felt that some of the content was becoming off-topic, especially the paragraph describing the exonic probe sets.

*We have refined the text in the introduction in light of these comments. It’s now shorter and more focused on metagenomic approaches.*

It would be beneficial to include a description of how the GridION adaptive sampling is implemented in the introduction. While the authors describe the requirements, there are no details on how the sequences are actually used.

*We have added some additional text to the Background section to make this clearer.*

There are a few places in the manuscript where there is the inappropriate contraction of words, e.g. "can't" 397, page 16

*These have been corrected.*

Reviewer 2:

This manuscript comprises both an evaluation of Nanopore adaptive sequencing in the context of bacterial metagenomics and the introduction and testing of a simple mathematical model to predict the degree of enrichment of any species in a community. Nanopore adaptive sequencing is an appealing technology in the context of metagenomics for the reasons outlined by the authors. In mixed communities being able to select for a species or strain of interest has numerous applications. The authors do not introduce a new bioinformatics strategy for doing this but instead evaluate the ONT algorithm. The major novelty in the study lies in the model and the results of testing seven species mock community algorithm which was tested over a range of fragment lengths.

The model is simple, being quite similar to the derivation of Michaelis-Menten kinetics for enzymes but appears to be very accurate. I believe that people performing such studies will find being able to predict the degree of enrichment of the target species to be very useful.

*We thank the reviewer for taking the time to read the manuscript and for their helpful comments. We hope we have been able to address all their comments in this new revision.*

I was less impressed by the mock community experiments themselves, seven species is not a complex community and may not be very representative of real samples not least because there was no strain diversity. Given that a lot of the value of this paper lies in the mock analysis I think a more realistic community would have been better. Furthermore, the authors worked from the assumption that the exact reference sequence of the strain of interest will be available. This is an unrealistic assumption. Many applications of the technique may consist of amplifying a species without knowing the precise strain that may be present. The authors could have explored this by including only genomes from related strains of the same species in the reference database. Or even if a larger taxonomic is targeted, for instance enriching for fungi in general would be very relevant in microbiome studies. It would have also added greatly to the paper if they had actually applied the method to a real sample in a real application context.

*This is a very similar comment to Reviewer #1 and we hope both reviewers will agree that we have addressed their comments in the new revision.*

*As we said above, in this new revision we have sequenced two additional samples using adaptive sequencing. In the first, we sequence a more complex gut microbial standard that includes 5 strains of E. coli. In the second, we sequence compost, a complex real-world sample. We show that enriching for a single strain of E. coli ends up enriching the other 4 strains as well. The desirability of this depends on the particular experiment being performed - sometimes this will be a problem, sometimes a benefit. We also show that good enrichment is achievable from the compost using both a previously published reference genome and a genome assembled from the same metagenomic sample.*

Some minor points are that I found the introduction a bit unfocussed the discussion of MAGs did not seem the obvious way to start a motivation of the method and the digression into RNA sequence also seemed a bit superfluous. It was also unclear what method of sequence comparison is used in the program tested here but that be because the details are not revealed by ONT.

*We’ve reworked the introduction in light of this comment. It’s now shorter and more focused on metagenomic approaches. Please see tracked changes.We now reference a comment on the nanopore community forum from an ONT employee that confirms minimap2 is used for sequence comparison.*

On balance whilst I believe the model is practical and of interest, the actual mock community results as performed do not sufficiently explore the real limitations of the method or clearly demonstrate practical uses of the methodology.

*We hope the two additional experiments described above address the reviewer’s reservations.*

**Second round of review**

**Reviewer 2**

The authors have put considerable effort into addressing the comments raised by myself and the other reviewer. They have added two additional experiments, a more complex mock community from Zymo and a real compost sample. These definitely go a long way to addressing our comments. It was interesting to see that in the presence of strain diversity all strains are enriched in the case of E coli. That is not surprising if enrichment is based on minimap. It is also arguably beneficial, you will rarely know precisely what strain is present (except perhaps in disease outbreaks) and you would want to enrich all relatives of the target. I feel though that some statistics were missing here, or possibly present but I missed them. That would be regarding the fraction of genome covered of the target and off target E coli strains. It is likely that parts of the pan genome in the off target strain may be missing. Similar to the effect observed for the compost genome. If the authors can add something to this effect then I will feel my comments have been addressed. I do not need to re-review the Manuscript. In case it is there and I missed it, just emphasise in the Discussion section.

**Authors Response**

**Point-by-point responses to the reviewers’ comments:**

The authors have put considerable effort into addressing the comments raised by myself and the other reviewer. They have added two additional experiments, a more complex mock community from Zymo and a real compost sample. These definitely go a long way to addressing our comments. It was interesting to see that in the presence of strain diversity all strains are enriched in the case of E coli. That is not surprising if enrichment is based on minimap. It is also arguably beneficial, you will rarely know precisely what strain is present (except perhaps in disease outbreaks) and you would want to enrich all relatives of the target. I feel though that some statistics were missing here, or possibly present but I missed them. That would be regarding the fraction of genome covered of the target and off target E coli strains. It is likely that parts of the pan genome in the off target strain may be missing. Similar to the effect observed for the compost genome. If the authors can add something to this effect then I will feel my comments have been addressed. I do not need to re-review the Manuscript. In case it is there and I missed it, just emphasise in the Discussion section.

*We thank the reviewer for looking at the latest version of the manuscript. They are correct that we hadn't included stats on the genome fraction, so we have now added these to the Results section of the paper.*
